# Disease-specific quality of life after septoplasty and bilateral inferior turbinate outfracture in patients with nasal obstruction^☆^

**DOI:** 10.1016/j.bjorl.2017.07.001

**Published:** 2017-07-29

**Authors:** Lucas Resende, Carolina do Carmo, Leão Mocellin, Rogério Pasinato, Marcos Mocellin

**Affiliations:** Universidade Federal do Paraná (UFPR), Hospital de Clínicas, Serviço de Otorrinolaringologia, Curitiba, PR, Brazil

**Keywords:** Nasal surgical procedures, Turbinates, Quality of life, Nasal septum, Nasal obstruction, Procedimentos cirúrgicos nasais, Cornetos, Qualidade de vida, Septo nasal, Obstrução nasal

## Abstract

**Introduction:**

Septal deviations might cause nasal obstruction and negative impact on the quality of life of individuals. The efficacy of septoplasty for treatment of septal deviation and the predictors of satisfactory surgical outcomes remain controversial. Technical variability, heterogeneity of research samples and absence of a solid tool for clinical evaluation are the main hindrances to the establishment of reliable statistical data regarding the procedure.

**Objective:**

To evaluate the clinical improvements in the disease-specific quality-of-life between patients submitted to septoplasty with bilateral outfracture of the inferior turbinate under sedation and local anesthesia in a tertiary hospital and to assess possible clinical–epidemiological variables associated with functional outcome.

**Methods:**

Fifty-two patients consecutively submitted to septoplasty with bilateral outfracture of the inferior turbinate for treatment of nasal obstruction filled in forms regarding clinical and epidemiological information during enrollment and had their symptom objectively quantified using the Nose Obstruction Symptom Evaluation (NOSE) scale preoperatively and one and three months after the procedure. Statistical analysis aimed to determine overall and stratified surgical outcomes and to investigate correlations between the clinical–epidemiological variables with the scores obtained.

**Results:**

Statistically significant improvement in the preoperative NOSE questionnaire compared to the scores obtained three months after surgery was demonstrated (*p* < 0.001, *T*-Wilcoxon), with strong correlation between the preoperative score and the postoperative improvement during this period (*r* = −0.614, *p* < 0.001, Spearman). After one month, patients reached in average 87.15% of the result obtained at the study termination. Smokers and patients with rhinitis and/or pulmonary comorbidity showed increased average preoperative NOSE scores, although without statistical significance (*p* > 0.05). Gender, age, history of rhinitis and presence of pulmonary comorbidity did not influence significantly surgical outcomes (*p* > 0.05). Smokers presented greater reduction in NOSE scores during the study (*p* = 0.043, *U*-Mann–Whitney).

**Conclusion:**

Septoplasty with bilateral outfracture of the inferior turbinate has proven to significantly improve disease-specific quality-of-life and this favorable outcome seems to occur precociously.

## Resumo

**Introdução:**

Os desvios septais podem causar obstrução nasal e impacto negativo na qualidade de vida dos indivíduos. A eficácia da septoplastia para o tratamento do desvio septal e os preditores de resultados cirúrgicos satisfatórios continuam controversos. A variabilidade técnica, a heterogeneidade das amostras de estudo e a ausência de uma ferramenta sólida para avaliação clínica são os principais obstáculos ao estabelecimento de dados estatísticos confiáveis sobre o procedimento.

**Objetivo:**

Avaliar a melhora clínica na qualidade de vida específica da doença entre pacientes submetidos a septoplastia e fratura bilateral da concha inferior sob sedação e anestesia local em um hospital terciário e possíveis variáveis clínico-epidemiológicas associadas ao desfecho funcional.

**Método:**

Cinquenta e dois pacientes consecutivamente submetidos a septoplastia e fratura bilateral da concha inferior para o tratamento da obstrução nasal preencheram formulários com informações clínicas e epidemiológicas durante a inclusão no estudo e tiveram seus sintomas quantificados objetivamente utilizando a escala de Avaliação de Sintomas de Obstrução Nasal (*Nose Obstruction Symptom Evaluation* - NOSE) no pré-operatório e um e três meses após o procedimento. A análise estatística objetivou determinar resultados cirúrgicos globais e estratificados e investigar correlações entre as variáveis clínico-epidemiológicas e os escores obtidos.

**Resultados:**

Foi demonstrada uma melhora estatisticamente significativa nos escores obtidos no questionário NOSE três meses após a cirurgia (*p* < 0,001, T-Wilcoxon), quando comparado com os escores obtidos no pré-operatório, com uma forte correlação entre o escore pré-operatório e a melhora pós-operatória durante esse período (r = -0,614, *p* < 0,001, Spearman). Após um mês, os pacientes atingiram em média 87,15% do resultado obtido ao término do estudo. Fumantes e pacientes com rinite e/ou comorbidade pulmonar apresentaram valores médios pré-operatórios do escore NOSE aumentados, embora sem significância estatística (*p* > 0,05). Sexo, idade, história de rinite e presença de comorbidade pulmonar não influenciaram significativamente os resultados cirúrgicos (*p* > 0,05). Os fumantes apresentaram maior redução nos escores de NOSE (*p* = 0,043, U-Mann-Whitney).

**Conclusão:**

A septoplastia e fratura bilateral da concha inferior demonstraram melhorar significativamente a qualidade de vida específica da doença e este resultado favorável parece ocorrer de forma precoce.

## Introduction

The nasal septum is a midline structure responsible for giving the nose its central position. From anterior to posterior it could be divided into three different portions, according to its composition: a membranous, a cartilaginous and a bony portion. When significantly deviated, it might result in important functional and esthetic problems. Nasal obstruction is the most common complaint in rhinological practice and septal deviation is considered its main cause. It has been estimated that up to one third of the general population presents with some degree of nasal obstruction, of which up to 25% are eligible for surgical procedures as part of the treatment.^1^

Indication for septoplasty is usually made on clinical grounds, although it might be supported by complementary evaluation.^2^ In general, the surgical approach is elected after failure of clinical treatment of nasal obstruction with medications such as topical corticosteroids, antihystaminics and descongestionants.^1^

The efficacy of septoplasty for treatment of septal deviation and the presence of predictors of satisfactory surgical response remain controversial in the literature. Technical variability, the heterogeneity of research samples and the absence of a solid tool for clinical evaluation of the patients are the main hindrances to the establishment of reliable statistical data. As a result, clinical outcome following septoplasty is still rather unpredictable.

Due to the great amount of individuals with symptomatic septal deviation, it is essential to continuously evaluate the efficacy of the different techniques of septoplasty.^3^ Previous studies have suggested objective benefits of outfracture in the treatment of nasal obstruction, with or without concomitant septoplasty.^4^, ^5^ However, data regarding subjective improvements after the procedure or preoperative predictive factors of better surgical outcomes remain scarce.

Many diagnostic tools, such as computed tomography, rhinomanometry, rhynometry and quality of life questionnaires, have been investigated to predict subgroups of patients with greater probability of having satisfactory outcomes after septoplasty. Stewart et al.^6^ developed and validated the Nose Obstruction Symptom Evaluation (NOSE) scale as a disease-specific quality-of-life questionnaire in nasal obstruction. It consists of a five-item questionnaire. For each item the patient score from zero to four. The sum of the items is then multiplied by five, resulting in a final score of the clinical burden associated with nasal obstruction which ranges from zero to a hundred. Table 1 shows the original version of this research tool.^6^, ^7^

**Table 1:** Nasal Obstruction Symptom Evaluation (NOSE) questionnaire.

Over the past one month, how much of a problem were the following conditions for you?					
	Not a problem	Very mild problem	Moderate problem	Fairly bad problem	Severe problem
Nasal congestion and stuffiness	0	1	2	3	4
Nasal blockage or obstruction	0	1	2	3	4
Trouble breathing through nose	0	1	2	3	4
Trouble sleeping	0	1	2	3	4
Unable to get air through nose during exercise	0	1	2	3	4

This is a prospective study, whose included patients agreed with the informed consent form, which was authorized by the Ethics Committee of The Hospital (n° 51009815.0.0000.0096). The primary goal of this study is to evaluate patients who were submitted to septoplasty with bilateral outfracture of the inferior turbinate in a tertiary Brazilian hospital in order to treat symptomatic septal deviation with or without hypertrophy of the inferior conchae, as to improvements in the disease-specific QL, measured by the NOSE questionnaire. Secondary goals included: to assess the correlation between the pre-operative NOSE scores and the score variation after three months of the study and to determine possible clinical–epidemiological variables associated with functional outcome of the procedure.

## Methods

The patients were consecutively recruited between May of 2015 and January of 2016. Inclusion criteria were: individuals with chronic nasal obstruction caused by septal deviation; persistent symptoms for over twelve weeks; failure of clinical treatment of eventually concomitant rhinitis; surgical indication of septoplasty and age above 18 years.

We excluded pregnant women, patients undergoing septoplasty as a surgical access to other sites or along with rhinoplasty and patients with history or diagnosis of other rhinological comorbidities such as septal perforation, craniofacial malformations, adenoid hypertrophy, granulomatosis and chronic rhinosinusitis (according to the EPOS 2007^8^ criteria). We also excluded patients with inferior conchae hypertrophy refractory to the use of topical vasoconstrictors and therefore with formal indication of surgical techniques including resection of redundant tissue.

Septoplasty was defined as an open surgery which aimed to straighten the nasal septum and a submucosal approach was preferred. In our study, this procedure was carried out under sedation (an initial 1 mcg/kg loading dose of midazolam in 10 min followed by continuous infusion of dexmedetomidine 0.2–0.7 mcg/kg/h) and local anesthesia with lydocaine with vasoconstrictor (1:100,000). The septum was therefore straightened after elevation of a mucoperichondrial and mucoperiosteal flap. This flap was raised after a septocolumellar or transfixating incision on the concave side of the deflection. After an initial chondrotomy adjacent to the deviated portion, a contralateral flap was also elevated. The removal of the bony and cartilaginous deviated areas was then performed, along with excision of any spurs and ridges. A careful approach was adopted in order to preserve the structure of the keystone area, which is paramount to the maintenance of the nose structure. If there was a caudal septal deflection, it was repositioned in midline and then anchored with U-sutures using the Metzembaum technique, originally described in 1990.^9^ A drainage incision and a transseptal suture along the area where the deviation had been removed were made to prevent hematoma formation. The bilateral turbinectomy was routinely performed as a simple, quickly and rather tolerable procedure. This procedure began with infiltration of both inferior turbinates with the same 1:100,000 lydocaine/epinephrine solution. Then, using a freer elevator, the entire turbinate was first moved medially and superiorly until a crunching sound was heard. Using the same elevator, the whole inferior turbinate was then moved inferiorly and laterally. By holding a large or medium Killian's nasal speculum vertically, each of its blades was inserted in each nostril, and the speculum was forcefully opened to achieve effective and full lateralization of both of the inferior turbinates. The use of postoperative splints or nasal packing was not necessary. The surgeries were performed by resident ENT physicians from our department, according to the evaluation and technical supervision from an attending physician. During the study, the patients were not using any medications, besides opportune treatment for rhinitis if necessary and tailored to the disease severity, according to ARIA (Allergic Rhinitis and its Impact on Asthma)^10^ guidelines.

The primary outcome was the disease-specific QL score, measured by the NOSE questionnaire validated for the Portuguese language, applied before surgery and one and three months after the procedure. Data analysis was carried out using the SPSS 10.0 (SPSS Inc., Chicago, IL). The non-parametric Wilcoxon *T* test was performed in order to compare the pre- and postoperative NOSE questionnaire scores obtained during the follow up. The Spearman's correlation coefficient was used to assess the correlation between the preoperative score and the postoperative improvement, calculated by the difference between postoperative (three months after surgery) and preoperative scores. Comparative analysis between subgroups according to their baseline characteristics was evaluated using the Mann–Whitney *U* test. A *p*-value lower than 5% was deemed significant.

## Results

**Figure 1:**
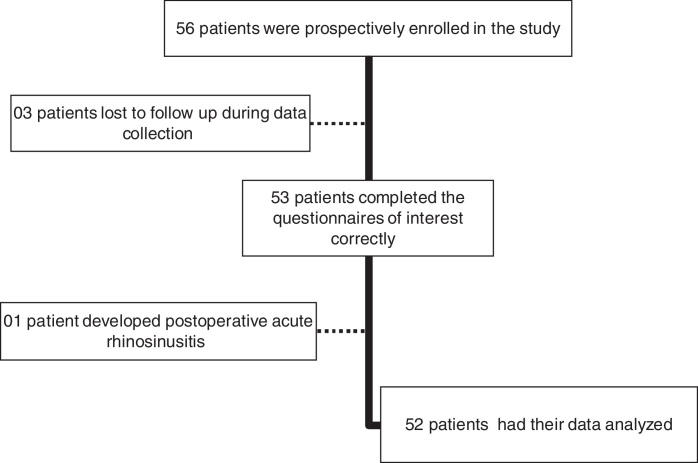
Study flow chart.

Fifty-six patients met the inclusion criteria. From these, three lost follow-up and had to be excluded. One patient had an acute episode of rhinosinusitis postoperatively and was also excluded from the study. As a result, 52 patients completed the study protocol and had their data included in statistical analysis (Fig. 1).

Clinical and epidemiological baseline characteristics and the average preoperative NOSE score for each subgroup are presented in Table 2. Descriptive statistical analysis of these data showed a negative impact of the following factors on preoperative NOSE scores: smoking, history of rhinitis, pulmonary comorbidity, bilateral symptoms and previous nasal trauma. However, none of these variables reached statistical significance (*p* > 0.05, *U* Mann–Whitney).

**Table 2:** Average NOSE score according to clinical and demographic variables, according to time of evaluation.

			Average NOSE score		
Variable	*n* (%)	Preoperative	After 1 month	After 3 months	Total
*Gender*					
Women	32 (61.54)	75.16	30.94	21.09	42.39
Men	20 (38.46)	74.75	27.00	24.25	42.00

*Age (years)*					
<30	17 (32.69)	74.11	22.65	15.59	37.45
30–54	28 (53.85)	74.82	31.07	27.14	44.34
>55	07 (13.46)	77.86	39.28	19.28	45.48

*Smoking*					
Yes	05 (9.62)	82.00	15.00	06.00	34.33
No	47 (90.38)	74.26	30.96	24.04	43.08

*Nasal trauma*					
Yes	05 (9.62)	85.00	30.00	27.00	47.33
No	36 (69.23)	75.97	28.88	22.08	42.31
Could not recall	01 (1.92)	70	45	55	56.66
Not investigated	10 (19.23)	67	29.5	17.5	38

*Pulmonary disease*					
Yes	08 (15.38)	83.13	40.00	24.37	49.17
No	44 (84.62)	73.52	27.50	21.93	40.98

*Allergic rhinitis*					
Yes	34 (65.38)	77.06	31.32	23.68	44.02
No	18 (34.52)	71.11	25.83	19.72	38.88

*Obstruction*					
Unilateral	29 (55.76)	72.24	–	–	29 (55.76)
Bilateral	23 (44.23)	78.47	–	–	23 (44.23)

*Total*	52	74.81	28.33	21.48	

**Figure 2:**
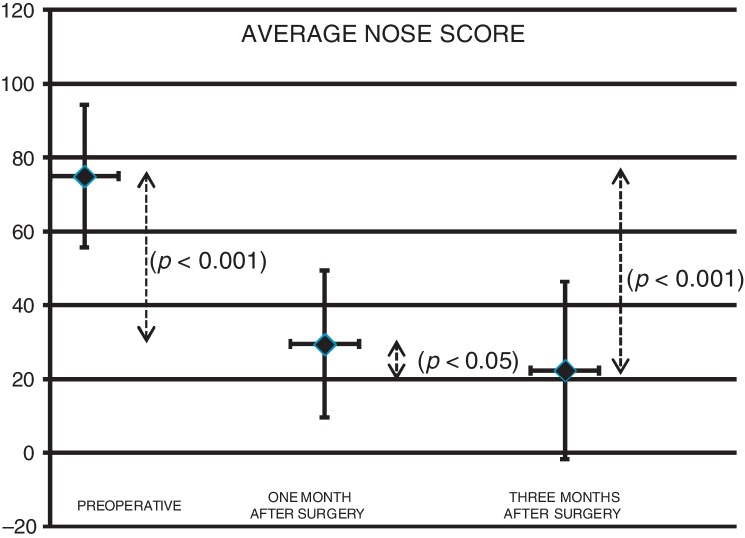
Preoperative and postoperative (one and three months) average NOSE scores (*p* < 0.05).

**Figure 3:**
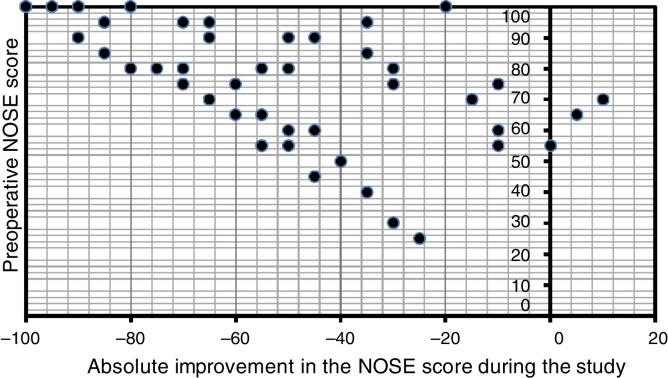
Scatter plot showing correlation between preoperative NOSE scores and the improvement in the score after 3 months of the surgery, calculated by the difference between preoperative NOSE values and the scores obtained at the study endpoint (*r* = −0.614, *p* < 0.001).

There was a statistically significant improvement assessed by the Wilcoxon test on the overall NOSE scores after three months of surgery, when compared to baseline data (*p* < 0.001). The reduction on the scores was also significant when comparing preoperative and one-month follow-up values (*p* < 0.001) and likewise when evaluating the scores after one and three months of the procedure (*p* = 0.0096). Fig. 2 depicts the progression of average NOSE scores during the study. Considering the mean NOSE value, it might be observed that 87.15% of the overall surgical outcome could already be noted at one-month follow-up. Spearman's correlation coefficient showed strong correlation between preoperative NOSE questionnaire and the subjective improvement reported (NOSE score reduction, *i.e*. the difference between the values of baseline NOSE score and of the same variable after three months of the surgery) (*r* = −0.614, *p* < 0.001) A scatter plot clearly depicts this correlation between preoperative NOSE scores and the magnitude of the improvement observed at the study endpoint (Fig. 3).

Mann–Whitney *U*-test did not show any statistically significant difference in the overall magnitude of reduction of initial NOSE scores at the study endpoint when the patients were grouped according to gender, age, associated rhinitis, type of symptom (if unilateral or bilateral), presence of pulmonary comorbidity or history of nasal trauma (*p* > 0.05) (Table 3). In this sample, patients who smoked showed significantly greater improvement in their baseline scores when compared to non-smoking patients (*p* = 0.043) (Table 3).

**Table 3:** *p*-Value obtained upon analysis of the impact of different clinical and epidemiological factors on the reduction of preoperative NOSE score after three months of the procedure.

Variable	*p*-Value
*Gender*	0.862

*Age group*	
Between the youngest and the intermediary groups	0.307
Between the intermediary and the oldest groups	0.472
Between the youngest and the oldest groups	0.844

*Smoking*	**0.043**
*Nasal trauma*	0.691
*Pulmonary disease*	0.213
*Type of obstruction (unilateral or bilateral)*	0.183
*Report of rhinitis*	0.763

Mann–Whitney *U*-test. Significance level < 0.05.

The estimated time of the whole procedure ranged from 15 to 45 min, with an average duration of about 25 min. Although not systematically counted and registered, the mean time of the turbinectomy alone was unlikely to exceed 5 min.

## Discussion

The surgical manipulation of the nasal septum for treatment of airflow obstruction has long been performed. The first report of a septoplasty dates back to 3500 b.c. in Egypt and it is documented in the *Ebers Papyru*. There are many described techniques and incisions for septal surgery, each one with its own advantages and drawbacks.^11^ Traditionally, septal deviation is surgically treated as an open procedure, with hemitransfixation of the anterior nasal mucosa and elevation of bilateral mucoperichondrial flaps. The bony and/or cartilaginous part of the septum implicated on the deviation is then selectively removed. Maximum tissue resection has been historically advocated in order to assure septal realignment. However, more aggressive approaches might impair future procedures and increase the risk of postoperative esthetical complications. Hence, avant-guarde techniques, such as the use of scarifications, have emerged with the intention to preserve the septal structure as much as possible.^3^

During the past fifteen years, a large number of studies aiming to evaluate the success rates of septoplasty were published. The results have been mixed, with positive rates ranging from 27% to 84% at a follow-up from 6 months to 11 years after surgery. These studies show different methodology for assessing clinical response, as well as diverse sample sizes and dropout rates. As a result, their findings cannot be compared. Some research has also been made on predictors of satisfactory post-septoplasty outcomes. Nonetheless, standardized guidelines for selecting patients with greater probability of surgical success are still lacking.^12^

Several recent papers have used NOSE questionnaire to investigate nasal surgery overall success.^7^, ^13^, ^14^ This questionnaire has been proved suitable for evaluating French and English-speaking populations.^9^ As for the factors affecting postoperative outcomes, one study has suggested that age might have an impact on clinical response.^15^ In another study, allergic rhinitis has also been found to predict worse outcomes.^14^

NOSE questionnaire has recently been validated for Portuguese-speaking patients.^16^ Preliminary studies have used this tool in order to corroborate subjective clinical improvement after septoplasty and to assess the success rates of specific surgical techniques.^17^, ^18^ Bezerra et al. have shown significative NOSE score reduction after three months of septoplasty with or without turbinectomy. The authors also demonstrated a strong correlation between preoperative scores and the magnitude of surgical response. In this study, gender did not interfere with postoperative outcomes.^18^

The present study confirmed the hypothesis that septoplasty with bilateral outfracture of the inferior turbinates improves disease-specific quality of life, measured by NOSE questionnaire after 3 months of the procedure. There was a statistically significant effect on NOSE average score during the study follow-up period (*p* < 0.001) (Fig. 2) and also a statistical correlation between the improvement in postoperative score and the preoperative score in the NOSE questionnaire (*r* = −0.614, *p* < 0.001) (Fig. 3).

It was also detected a significant improvement on NOSE scores also when comparing the preoperative values with those obtained during the one month follow-up visit (*p* < 0.001) and between the scores obtained after one and three months of the surgery (*p* < 0.05) (Fig. 2). It should be highlighted that in this sample the major improvement on average NOSE scores during the follow-up was observed after one month of the surgery (87.15% of the reduction of the score) (Table 2). These data indicate that the postoperative impact on quality of life might be found at least up to 3 months after surgery, although the substantial benefit of septoplasty with outfracture of inferior turbinates could be already determined 30 days after the procedure.

The subgroups of smoker patients and of those with history of previous nasal trauma, comorbid lung illnesses or concomitant rhinitis showed comparatively worse average preoperative NOSE scores, although these results did not reach statistical significance (*p* > 0.05).

In this study, no significant difference was observed between the improvements on NOSE scores stratified by gender, age, concomitant rhinitis, comorbid lung disease, history of nasal trauma or quality of nasal obstruction (if unilateral or bilateral) (*p* > 0.05). In this sample, smokers showed a significantly greater improvement on average NOSE scores (*p* = 0.043) (Table 3).

The study had the following limitations: lack of a control group, non-randomized sample and the fact that it was conducted in a tertiary referral center. Being held in a tertiary hospital might have given rise to a selection bias, but provided homogeneity of intervention in terms of operative team involved, surgical techniques employed and postoperative follow-up. The lack of an alternative treatment with proven efficacy for refractory nasal obstruction affected the feasibility of using a control group.

The size of the sample, despite its suitability for investigating the primary outcome of the study, might hamper the analysis of the results according to subgroups of patients. Hence, data obtained after stratifying the cohort must be further confirmed by larger studies.

Objective methods for assessment of nasal obstruction were not used in this study. A review of the current literature reveals that there is a poor correlation between objective anatomical data (grades of septal deviation or hypertrophy of inferior turbinates) and the scores obtained on disease-specific quality of life questionnaires. Therefore, acoustic rhinometry must provide a geometric representation of the nasal cavity, but with poor clinical correlation.^16^, ^19^, ^20^

The effectiveness of septoplasty for refractory nasal obstruction has been previously demonstrated using different research tools, both qualitative and quantitative.^16^, ^21^, ^22^, ^23^, ^24^ However, in Brazil, only Bezerra et al. had published their data on surgical outcomes using a specific quality of life questionnaire. Other Brazilian studies had evaluated different turbinectomy techniques.^25^, ^26^ Nevertheless, this study sought to assess a specific, standardized and combined technique, which was systematically employed to all included patients undergoing septoplasty, regardless of the grade of inferior turbinates hypertrophy. However, it should be outlined that we excluded patients with inferior turbinates hypertrophy refractory to topical decongestants, as these individuals needed to undergo different procedures which would warrant excision of redundant tissue. Due to the great variability of approaches to the inferior turbinates during septoplasty, studies focusing on investigation of standardized techniques can minimize a strong bias. We did not assess the grade of the septal deviation. This fact does not have an impact on the main conclusions of the study, if we consider that they represent the overall results we would expect for a heterogeneous universe of patients, similar to that we routinely found in clinical practices and tertiary hospitals. However, we highlight that not evaluating the grade of septal deviation might have influenced the statistical data obtained when comparing the subgroups of the sample. It should also be emphasized that, even when carried out systematically, bilateral inferior turbinate outfracture did not increase morbidity among these individuals, as the overall complication rate in the study was low (one patient developed postoperative acute rhinosinusitis and there was no incidence of abnormal bleeding requiring nasal packing or revision surgery). The absence of increased morbidity was fortunately accompanied by maintenance of great subjective outcomes. However, as turbinectomy was performed along with correction of the septal deviation, the conclusions of this study cannot be extrapolated to evaluate outfracture of the inferior turbinates without septoplasty. Yet, there is still no consensus regarding the long-term efficacy of inferior turbinates outfracture and our study was capable of predicting the clinical outcome only up to three months after surgery. Longterm follow-up studies with larger cohorts might overcome these potential limitations and help confirm the results we obtained.

## Conclusion

Patients undergoing septoplasty with bilateral outfracture of the inferior turbinate under sedation and local anesthesia significantly improved disease-specific quality-of-life, measured by NOSE questionnaire. There was a strong correlation between worse preoperative scores and the magnitude of reduction of NOSE scores at the termination of the study. Moreover, the scores continued to diminish up to the third month of follow-up, but a large part of the improvement achieved was observed within the first month after surgery. Apart from smoking, no other clinical or epidemiological characteristic seems to have an impact on postoperative outcomes.

## Conflicts of interest

The authors declare no conflicts of interest.
